# Vulvar dermatofibrosarcoma protuberans, an unusual anatomical location

**DOI:** 10.1093/bjrcr/uaaf030

**Published:** 2025-05-29

**Authors:** Bano Alsaleh, Ahmed Alanzi, Mohamed Alsaleh, Ahmed Alsaleh, Fouad Aladel

**Affiliations:** Radiology Department, King Hamad University Hospital, Busaiteen, 24343, Kingdom of Bahrain; Anaesthesia and Pain Management Department, King Hamad University Hospital, Busaiteen, 24343, Kingdom of Bahrain; Pediatric Department, Mohammed Bin Khalifa Bin Salman Al Khalifa Specialist Cardiac Center, Al Riffa, 28743, Kingdom of Bahrain; Royal College of Surgeons in Ireland—Bahrain, Adliya, 15503, Kingdom of Bahrain; Radiology Department, King Fahad Specialist Hospital, Dammam, 15215, Kingdom of Saudi Arabia

**Keywords:** Gynaecology, vulvar neoplasm, dermatofibrosarcoma protuberans, cutaneous sarcoma, MRI of vulva, diagnostic radiology, mass

## Abstract

Vulvar dermatofibrosarcoma protuberans (DFSP) is a rare pathology. So far, only limited number of cases have been reported in literature. In the present case, we discuss a 38-year-old female presented with a painful left vulvar mass. She had a prior history of a left vulvar mass excision which was histopathologically confirmed as benign spindle cell epithelioma. The current mass, extending from the left labia majora to the left gluteal fold, was assessed via contrast-enhanced magnetic resonance imaging (MRI), revealing a well-defined, lobulated lesion with proximity to the distal urethra and clitoris without definite invasion. The patient underwent a wide local excision, radical vulvectomy, and left inguinofemoral lymphadenectomy. Postoperatively, she experienced fever, vulvar swelling, and dysuria. Follow-up MRI demonstrated total resolution of the vulvar mass and collection with no recurrence. Histopathology identified the mass as DFSP, with all surgical margins negative.

## Background

The vulvar dermatofibrosarcoma protuberans (DFSP) is a slow-growing tumour that rarely metastasizes, with distant spread occurring in less than 5% of cases.^1^ In 1924, Darier and Ferrand^2^ first described a vulvar DFSP case. DFSP typically arises from the dermis and then extends into underlying subcutaneous tissues. The incidence of local recurrence is common in DFSP, occurring in 20% to 50% of cases.^3^ Due to its gradual progression, diagnosis often occurs at an advanced stage. The diagnosis of vulvar DFSP is particularly challenging as it is often confused with other tumours. The preferred approach for both initial diagnosis and recurrence is radical surgery to achieve complete excision with clear surgical margins.^1^ In this case report, we discuss a case of rare vulvar DFSP.

## Case presentation

A 38-year-old female (P1 + 0) presented with a painful vulvar mass. Her prior medical history revealed an excision of left vulvar mass, which was confirmed as benign mixed tumour (spindle cell epithelioma) by histopathology. However, the tumour recurred a year later. She reported no weight loss, appetite changes, intermenstrual bleeding, or abnormal vaginal discharge, and her menstruation cycle was regular. On examination, a tender mass was noted on the left side of the vulva, extending towards the mons pubis; however, no overlying skin redness, or nodularity was observed. She has a history of a twin pregnancy 4 years ago, delivered via normal vaginal delivery.

## Investigations

**Figure 1.**
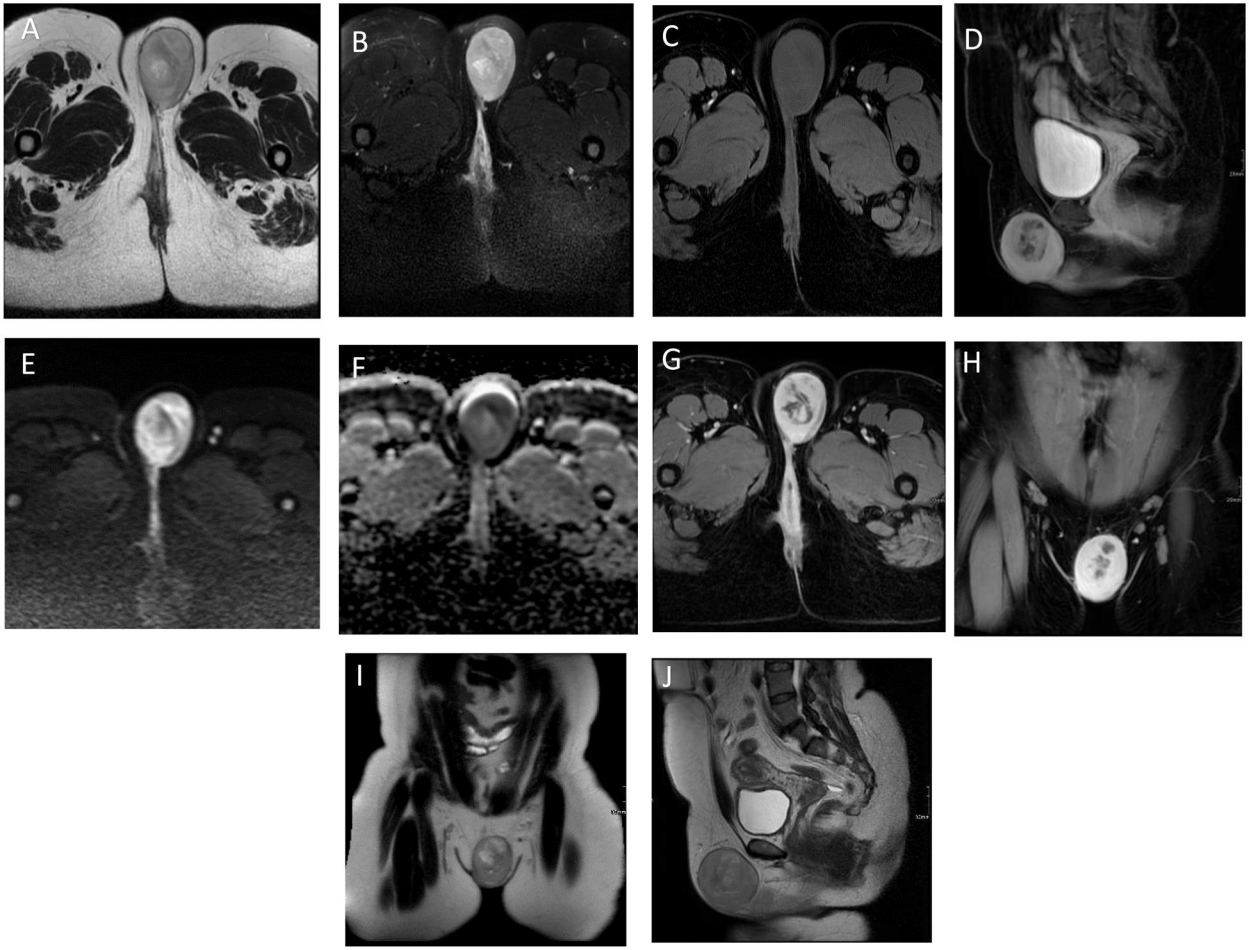
Multiple selected images from different sequences of the contrast-enhanced MRI of the pelvis, displayed in axial, coronal, and sagittal planes, showing: left-sided subcutaneous lobulated vulvar mass measuring 5.8 × 6.8 cm that extends to the labia majora and more inferiorly towards the left gluteal fold (image I), with close proximity to the distal urethra and clitoris without obvious invasion. The mass exhibits a smooth contour, intermediate signal intensity (SI) on T2-weighted images (images A, I, J) with no fat suppression on T2/STIR (image B), low SI on T1-fat saturated images (image C), diffusion restriction (image E, F), and intense progressive enhancement in the post-contrast sequences (images B, D, H).

Contrast-enhanced magnetic resonance imaging (MRI) of the pelvis revealed a large well-defined lobulated vulvar mass (6.8 cm) extending along left labia majora and inferior towards left gluteal fold, which has close proximity to the distal urethra and clitoris without definite invasion (Figure 1).

## Treatment

The patient underwent wide local excision with radical vulvectomy and resection of the margin, confirmed by frozen section, along with left Inguinofemoral lymphadenectomy. During her post-operative stay, she complained of fever (38.5), left vulvar swelling and dysuria for 1 day. Examination revealed significant swelling and severe tenderness in the left groin, accompanied by skin redness but without any wound gap or discharge. She underwent an ultrasound evaluation of the swollen soft tissues, which showed a heterogenous collection, followed by a computed tomography (CT) scan of the pelvis showing mild peripheral wall enhancement with a central fluid density collection, likely a postoperative collection/hematoma with superimposed infection/abscess. The swelling opened spontaneously through a sinus with draining discharge. Jackson-Pratt (JP) drain was then placed and applied under negative pressure. She was then started on antibiotics. The discharge culture showed *Staphylococcus aureus*. Follow-up MRI showed total resolution of the vulvar mass and collection with no recurrence or residual.

## Outcome and follow-up

Histopathology of the excised left vulvar mass revealed DFSP, with all surgical margins negative. The left inguinal lymph node dissection revealed 9 lymph nodes, all of which were negative for malignancy (0/9).

## Discussion

DFSP typically involves local infiltration into the dermis and subcutaneous tissue, often extending into surrounding structures like fascia, muscle, and even bone.^1^ While DFSP commonly appears on the trunk (42%–62%) and extremities (16%–30%), with some cases on the head and neck (10%–16%), its occurrence in the vulva is extremely rare, with only around 70 cases reported to date.^1^^,^^3^ In our case, 38 years old female was presented with mass of vulva, which extended along the left labia majora. Labia majora (52.2%) is the most common site of presentation in vulvar DFSP, followed by mons pubis (11.6%).^3^ Nguyen et al.^4^ in their systematic review that included 54 patients with vulvar DFSP, reported that labia majora was the site of presentation in 53.7% of cases. In the present case, the patient did not report intermenstrual bleeding, or abnormal vaginal discharge, which is consistent with the majority of the vulvular DFSP cases. However, in some rare cases (7.2%), bleeding, and dyspareunia have been reported in literature.^3^ For example, Neff et al.^5^ in their case study reported the bleeding vulvar mass. Grossly, the mass seems well-defined due to condensation of connective tissue at the margins of the nodules as seen in the present case.^5^

The behaviour of vulvar DFSP is similar to other DFSP localizations. Distant metastases are rarely observed while spontaneous local regressions are commonly found.^6^ For example, Ghorbani et al.^7^ in their case series presented 4 cases of vulvar DFSP. In one of these patients, the tumour recurred every 2 to 4 years for the next 20 years after the first surgery. Similarly, in a large care series of 13 patients by Edelweiss and Malpica, 7 patients had local recurrence whereas one patient had distant metastasis to the lungs.^8^ For proper management of vulvar DFSP, careful diagnosis of the tumour is crucial. MRI is effective in identifying the extent and location of DFSP. On MRI, the tumours are superficial, have a low signal on T1-weighted images, and appears hyperintense on T2-weighted images, especially in the central areas due to increased water content.^1^ The differential diagnosis is complicated as it is difficult to tell the difference between a DFSP and other fibrohistiocystic neoplasms. The differential diagnosis of vulvular DFSP includes leiomyosarcoma, which also consists of spindle-shaped cells. However, it can be distinguished immunohistochemically, as leiomyosarcoma typically shows positive staining for smooth muscle markers. Another uncommon tumour that may be confused with DFSP in the vulvar region is the solitary fibrous tumour. This tumour is characterized by a “patternless” arrangement of spindle cells, unlike the classic storiform pattern seen in DFSP.^1^ Recently, Yu et al.^9^ has reported that dynamic contrast-enhanced HR-MRI can show growth characteristics of DFSPs, and can serve as a tool for differential diagnosis of DFSP.

The definitive diagnosis of DFSP generally relies on histopathological examination and immunohistochemical analysis. However, molecular confirmation may also be helpful in complicated cases. For example, in a case reported by Wiszniewska et al., a 44-year-old woman was initially misdiagnosed with vulvar neurofibroma due to the absence of immunohistochemical findings and typical morphology. However, peripheral adipose tissue trapping led to suspicion of DFSP, which was confirmed by the detection of characteristic COL1A1/PDGFB fusion transcript by reverse-transcription polymerase chain reaction.^10^ MRI is commonly used to assess the depth of the tumour. In cases of positive surgical margins, MRI has a sensitivity of 60% and specificity of 100%. CT scan can be used if bone involvement is suspected.^11^

The treatment of vulvar DFSP involves the surgical excision of the tumour. As in the present case, the patient underwent wide local excision with radical vulvectomy and resection of the margins, confirmed by frozen section, along with left Inguinofemoral lymphadenectomy. Leake et al.^12^ have also argued in favour of frozen sections of margins to ensure complete resection of the tumour in their case series. Although wide local excision is currently adopted approach in most cases, Nguyen et al.,^4^ in their review reported that patients undergoing Mohs micrographic surgery had a lower risk of recurrence in cases of vulvular DFSP. The objective of surgery is to achieve 3 cm negative margin. Patients who have wide negative margins are less likely to have recurrence.^11^ After surgery, follow-up with MRI is essential at regular intervals for the next 3 years as the tumour is most likely to have a recurrence within this period. The overall survival rate of DFSP has been reported to be 91%–100%. The rates of reoccurrence have been reported to be 20%–40%.^13^

## Learning points

- DFSP is a slow-growing tumour with a high rate of local recurrence which can be a treatment challenge, but rarely metastasizes.
- DFSP should be considered in all skin lesions slowly invading the subcutaneous tissues.
- DFSP most commonly involves the trunk. The vulva is an unusual anatomical site of involvement, yet DFSP should not be overlooked in the differential diagnosis.
- Diagnosis can be made via MRI with IV contrast, which also aids in evaluating the extent of soft tissue involvement and in optimal pre-operative planning.
- Establishing diagnosis early is key to prevent aggressive local invasion to the adjacent soft tissues, ensure negative margins on wide resection, and to reduce the chance of local recurrence.
